# A Point Cloud Data-Driven Pallet Pose Estimation Method Using an Active Binocular Vision Sensor

**DOI:** 10.3390/s23031217

**Published:** 2023-01-20

**Authors:** Yiping Shao, Zhengshuai Fan, Baochang Zhu, Jiansha Lu, Yiding Lang

**Affiliations:** 1 College of Mechanical Engineering, Zhejiang University of Technology, Hangzhou 310023, China; 2 Noblelift Intelligent Equipment Co., Ltd., Huzhou 313100, China; 3 Ningbo Fujia Industrial Co., Ltd., Ningbo 330200, China

**Keywords:** driverless industrial trucks, pose estimation, adaptive Gaussian weight-based fast point feature histogram, point cloud registration

## Abstract

Pallet pose estimation is one of the key technologies for automated fork pickup of driverless industrial trucks. Due to the complex working environment and the enormous amount of data, the existing pose estimation approaches cannot meet the working requirements of intelligent logistics equipment in terms of high accuracy and real time. A point cloud data-driven pallet pose estimation method using an active binocular vision sensor is proposed, which consists of point cloud preprocessing, Adaptive Gaussian Weight-based Fast Point Feature Histogram extraction and point cloud registration. The proposed method overcomes the shortcomings of traditional pose estimation methods, such as poor robustness, time consumption and low accuracy, and realizes the efficient and accurate estimation of pallet pose for driverless industrial trucks. Compared with traditional Fast Point Feature Histogram and Signature of Histogram of Orientation, the experimental results show that the proposed approach is superior to the above two methods, improving the accuracy by over 35% and reducing the feature extraction time by over 30%, thereby verifying the effectiveness and superiority of the proposed method.

## 1. Introduction

**Figure 1:**
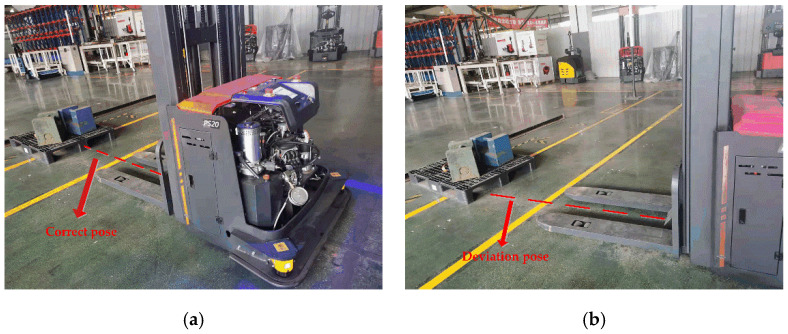
Diagram of pallet position deviation. (**a**) Correct pose. (**b**) Deviation pose.

The application of intelligent logistics equipment has played a key role in the transformation and upgrading of the manufacturing industry in recent years. Driverless industrial trucks are common intelligent logistics equipment [1] and widely used in warehousing, production, medical treatment, the service industry and other fields. It can realize automatic material handling and improve production efficiency and lower production costs for intelligent logistics systems [2]. However, as shown in Figure 1, due to the influence of obstacles, uneven lighting, human intervention and other factors, a certain deviation is caused between the actual pose and the correct pose of the pallet. As a result, collision and incomplete forks will occur when driverless industrial trucks forklift pallets, which will lead to dumping, damage and safety problems. Therefore, it is necessary to upgrade the technology of traditional driverless industrial trucks and introduce some external industrial vision sensors [3] to achieve the purpose of adaptive and automatic production.

In order to solve the problem shown in Figure 1, based on the data collected by the vision sensor, it is necessary to estimate the pose of the pallet when the driverless industrial truck is driven to a certain distance in front of the pallet, so as to correct the pose deviation of the pallet and ensure the efficiency and safety of the logistics process. There are two main kinds of target pose estimation methods: LIDAR based and vision based. The LIDAR-based methods are mature and have high estimation accuracy. Baglivo et al. [4] proposed an efficient scheme, which combined a laser range-based object localization approach with PC-Sliding. Mohamed et al. [5] presented a novel architecture allowing a robot to detect, localize and track pallets using machine learning techniques based on an onboard 2D laser rangefinder. Zhang et al. [6] improved the matching degree of multi-modal features of the target and achieved accurate target pose estimation by fusing the range of view, aerial view and RGB view of the LIDAR.

However, due to the problems of high cost, limited range and difficulty in eliminating cumulative errors of LIDAR-based methods, vision-based methods are often adopted for pose estimation in indoor environments with sufficient illumination. The vision-based pose estimation methods mainly include 2D vision and 3D vision. In certain work environments, the 2D vision-based methods have been widely used in the pose estimation of the target object [7,8,9,10]. Varga et al. [11] obtained the position and pose information of pallets by intensity image and a stereo camera, and the LBP featured descriptor was introduced to realize automatic fork picking of pallets. Monocular images and matching of 2D deformation patterns were used to estimate the pose of known objects in dynamic environments by Casado et al. [12]. With the rapid development of low-cost depth sensors, object pose estimation has been transformed from traditional single-point and segmented measurements to dense point clouds and full-profile measurements [13,14,15,16]. Compared with 2D vision, 3D vision can obtain one more dimension of target information, which solves the problem of information loss in the process of mapping from 3D space to 2D space and has gradually become a hot topic in current research [17,18]. The current popular 3D vision solution is to estimate the pose of the target by point cloud registration. Common point cloud registration algorithms include the Normal Distributions Transform (NDT) [19] algorithm, Principal Component Analysis (PCA) algorithm [20], Iterative Closest Point (ICP) algorithm [21], and many other improved algorithms. The principle of the traditional ICP algorithm is popular and easy to understand, and the registration effect is remarkable; thus, it is widely used in point cloud registration. However, the ICP algorithm also has drawbacks, such as long calculation time and inability to solve local optimal problems [22]. Wu et al. [23] proposed a novel nearest neighbor search algorithm to improve the iteration speed of point cloud registration. Fotsing et al. proposed a novel region growing-based approach [24] for plane detection in unorganized point clouds extract reliable seeds by the Iterative Closest Point algorithm to enhance the performance and the quality of the results.

In addition, the initial pose of the point cloud to be registered has an important influence on the accuracy of point cloud registration. The coarse registration based on feature matching is used to obtain a better initial pose, which is beneficial to improve the pose estimation accuracy [25]. Rusu [26] proposed the Fast Point Feature Histogram (FPFH) descriptor, which can be used to describe the neighborhood geometry information of the query point and is often used to estimate the target pose. SaltiS et al. [27] proposed a Signature of Histograms of Orientation (SHOT), which can represent topological features, has rotation invariance and is robust to noise. Li et al. [28] proposed real-time path planning based on a VFH feature descriptor, which was robust against a large degree of surface noise and missing depth information; however, pose estimation could fail when the object was placed symmetrically with the viewpoint. Compared with other feature descriptors, the FPFH has the characteristics of fast computation and high accuracy, and it is used to describe the geometric features of the pallet point cloud in this study. However, the current FPFH descriptor also has some drawbacks. Firstly, when selecting the weight coefficient of the FPFH, only the Euclidean distance between the query point and the neighborhood point is considered, which makes the weight order difference too large and reduces the robustness of the FPFH feature descriptor. In addition, the current calculation approach of the FPFH feature descriptor does not consider the selection criteria of neighborhood radius and is usually debugged based on experience to determine neighborhood radius, which reduces the efficiency and accuracy of pose estimation.

In view of the above problems, a pallet pose estimation approach based on an Adaptive Gaussian Weight-based Fast Point Feature Histogram is proposed. On one hand, when determining the neighborhood radius of the descriptor, the optimal neighborhood radius of each point is obtained based on the minimum rule of neighborhood feature entropy function, which overcomes the randomness of neighborhood radius parameters debugging manually. On the other hand, when determining the weight of neighborhood points, the weight calculation formula is redefined according to the average distance and standard deviation between key points and their neighborhood points, which makes the weight of each neighborhood point controlled within a certain range.

The remainder of the paper is organized as follows: In Section 2, an overview of the proposed approach and the specific steps of the method are described. In Section 3, two cases are presented to verify the effectiveness of the proposed approach in engineering application, and the experimental results are analyzed and discussed. Finally, a conclusion is drawn in Section 4.

## 2. The Proposed Pallet Pose Estimation Method

### 2.1. Overview of the Proposed Approach

**Figure 2:**
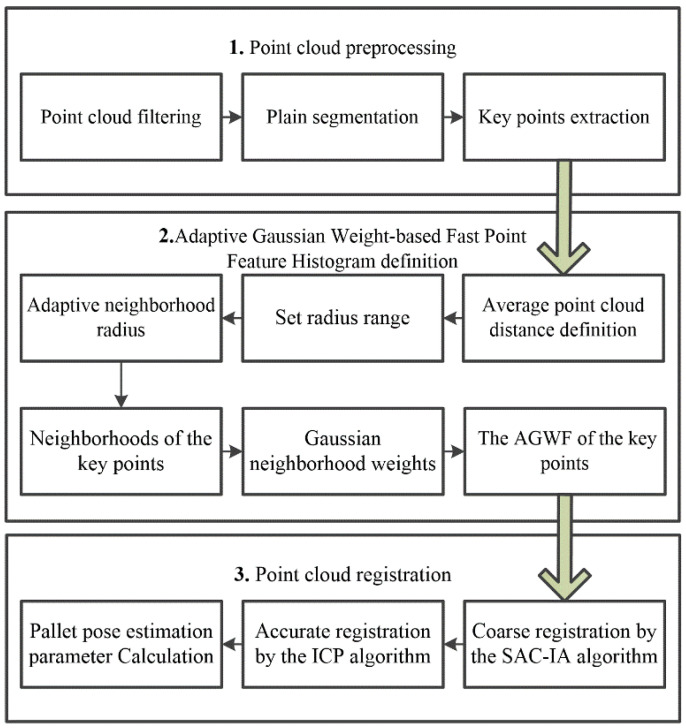
Flowchart of the pallet pose estimation method.

In order to realize the accurate position of pallets for driverless industrial trucks in the storage environment, a pallet pose estimation method based on an Adaptive Gaussian Weight-based Fast Point Feature Histogram is proposed; the procedure involves the following steps, and the flowchart is shown in Figure 2.

Step 1: Point cloud preprocessing. The source point cloud of the pallet and the scene point cloud containing the pallet are collected using an active binocular vision sensor. The redundant scene information in the scene cloud is removed through the pass-through filtering and voxel mesh downsampling method [29], and the plane segmentation algorithm is used to obtain the target pallet point cloud. The key points in the target point cloud are extracted by the Intrinsic Shape Signatures (ISS) algorithm [30].

Step 2: Adaptive Gaussian Weight-based Fast Point Feature Histogram definition. Adaptive optimal neighborhood radius is used to determine the neighborhood range of each point in the target point cloud. By calculating the mean value and variance of the distance between each key point and its neighborhood, the Adaptive Gaussian weight-based Fast Point Feature Histogram (AGWF) of each key point is obtained.

Step 3: Point cloud registration. According to the AGWF feature descriptor, the SAC-IA (sample consensus initial alignment) algorithm is used to coarsely register the source point cloud with the target point cloud. Then, the ICP algorithm is used to transform the point cloud iteratively and obtain the optimal rigid transformation matrix. The matrix parameters are converted into horizontal deviation and angle to realize pallet pose estimation.

### 2.2. Point Cloud Preprocessing

#### 2.2.1. Point Cloud Filtering

The original source point cloud P containing the pallet and the original scene point cloud Q_so_ (about millions of points) are collected by an active binocular vision sensor. The efficiency and accuracy of point cloud processing will be reduced due to the large number of acquired scene point clouds and a large amount of redundant scene information. The filtering interval is determined according to the spatial position relationship between the pallet and the driverless industrial trucks in the standard state, and the invalid point clouds and background information in the original scene point cloud Q_so_ are removed by the classical pass-through filter. A large number of redundant points are removed by voxel grid downsampling, and the complete geometric features of the point cloud are retained to obtain the filtered scene point cloud Q_s_.

The specific steps of the specific steps of voxel grid downsampling are as follows: (1) In point cloud Q_so_, the maximum and minimum values in X, Y and Z directions are x_max_, x_min_, y_max_, y_min_, z_max_, z_min_, respectively. Set the dimensions of the voxel grid d_0_, where $m=\left\lceil (x_{\max}-x_{\min})/d_{0}\right\rceil$,$n=\left\lceil (y_{\max}-y_{\min})/d_{0}\right\rceil$, $l=\left\lceil (z_{\max}-z_{\min})/d_{0}\right\rceil$, and ⌈⌉ represents round up. (2) Encode each point Q_v_(x_v_, y_v_, z_v_) in Q_so_ as (m_v_, l_v_, n_v_) to determine which grid each point belongs to, where $m_{v}=\left\lfloor (x_{v}-x_{\min})/d_{0}\right\rfloor$,$n_{v}=\left\lfloor (y_{v}-y_{\min})/d_{0}\right\rfloor$,$l_{v}=\left\lfloor (z_{v}-z_{\min})/d_{0}\right\rfloor$,and ⌊⌋ represents round down. If there are some points in a voxel grid, calculate its center of gravity C_0_ = (x_0_, y_0_, z_0_), and replace the points in each grid by the point nearest to the center of gravity to obtain the filtered scene point cloud Q_s_, where $x_{0}=\frac{1}{k}\sum_{i=1}^{k}x_{i}$,$y_{0}=\frac{1}{k}\sum_{i=1}^{k}y_{i}$,$z_{0}=\frac{1}{k}\sum_{i=1}^{k}y_{i}$, and k represents the number of points in the grid. 

#### 2.2.2. Plain Segmentation

Because the filtered scene point cloud Q_s_ still contains pallet, wall, ground and other information, and the pallet needs to be separated from the wall and ground, the plane segmentation method based on Random Sampling Consensus (RANSAC) is used to find the points belonging to the plane iteratively according to the set plane model. Meanwhile, the ground with different degrees of fluctuation can be detected by setting the model distance threshold. The specific steps are as follows: (1) The initial plane model A_x_ + B_y_ + C_z_ + D = 0 is constructed by selecting any three points from the filtered scene point cloud. (2) The distance d_i_ from point q_i_ (points in the point cloud Q_s_) to the initial plane and the angle α_i_ between the coordinates of point q_i_ and the normal vector of the initial plane are calculated. A distance threshold d_e_ and an angle threshold α_e_ are set. If d_i_ < d_e_ and α_i_ < α_e_, then point q_i_ is considered an in-plane point. (3) Iterations are carried out continuously until the number of in-plain points reaches the threshold t, and the final fitting plane model representing the wall and ground is removed, obtaining the target pallet point cloud Q.

#### 2.2.3. Key Points Extraction 

After filtering and plane segmentation, there are still tens of thousands of point clouds in the target pallet point cloud Q, which will reduce the pose estimation efficiency and fail to meet the requirements of the operation of driverless industrial trucks. Therefore, the points with obvious geometric features are selected from the target point cloud Q to form the key point set Q_t_, and only the features of the key points are extracted, which can significantly improve the efficiency of feature extraction of the point cloud. Due to the advantages of high speed, accuracy and robustness, an intrinsic Shape Signature (ISS) algorithm is developed to extract key points, and it is suitable for various applications [31]. The main steps are as follows: (1) Establish a local coordinate at each point q_v_ in point cloud Q and set a neighborhood search radius r_f_. (2) Search for the neighborhoods of the query point q_v_ with r_f_ and obtain their neighborhood points q_j_; the weight $w_{v}{}_{j}={\left(\left\|q_{j}-q_{v}\right\|\right)}^{-1}$ is calculated according to the Euclidean distance between q_v_ and q_j_. (3) Calculate the weighted neighborhood covariance matrix $C_{i}=\sum_{\left|q_{i}-q_{j}\right|<r_{frame}}w_{ij}(q_{i}-q_{j}){\left(q_{i}-q_{j}\right)}^{T}/\sum_{\left|q_{i}-q_{j}\right|<r_{frame}}w_{ij}$ of q_v_. (4) The eigenvalues $\lambda_{v}^{1},\lambda_{v}^{2}$ and $\lambda_{v}^{3}$ are obtained by eigenvalue decomposition of the covariance matrix, and they are arranged in descending order. (5) Set the thresholds $\varepsilon_{1}$ and $\varepsilon_{2}$$\left(\varepsilon_{1},\varepsilon_{2}\leq 1\right)$. If $\frac{\lambda_{1}^{2}}{\lambda_{v}^{1}}\leq \varepsilon_{1},\frac{\lambda_{1}^{3}}{\lambda_{v}^{2}}\leq \varepsilon_{2}$, and the points are supposed to be the key points, otherwise, iterate over the next point. Repeat the process until all the points have been traversed, and finally obtain the key point set $Q_{k}$ of the target point.

### 2.3. Adaptive Gaussian Weight-based Fast Point Feature Histogram Definition

#### 2.3.1. Adaptive Neighborhood Radius

The premise of accurate pose estimation is to construct a feature descriptor of the pallet point cloud with high efficiency, strong robustness and high accuracy; the neighborhood radius is an important factor affecting the performance of feature descriptors. The neighborhood radius of traditional FPFH is usually set to a fixed value according to experience, which reduces the speed of feature extraction. A neighborhood radius selection criterion based on adaptive neighborhood feature entropy is proposed to obtain the neighborhood radius of each key point q_k_ (points in the key point set Q_k_) adaptively. The detailed steps are as follows:

1. Set the range of point cloud neighborhood search radius r_j_ from lower limit r_min_ to upper limit r_max_ with radius interval r_d_. The upper and lower limits of the radius range are determined by the average point cloud distance d_p_, which is defined as follows [32]:
  (1) $$d_{p}=\frac{1}{N}\sum_{}^{}d_{m}$$
  where N represents the total number of points in the pallet point cloud, and d_m_ represents the distance of each key point q_k_ from its nearest point. Set $r_{\min}=d_{p}$, $r_{\max}=2d_{p}$, $r_{j+1}=r_{j}+\Delta r$, where j=1,2,….
2. Calculate the covariance matrix and eigenvalues corresponding to different neighborhood radius r_j_. The neighborhood covariance matrix M is defined as follows:
  (2) $$M=\left[\begin{array}{ccc} e_{1} & e_{2} & e_{3} \end{array}\right]\left[\begin{array}{ccc} \lambda_{1} & 0 & 0\\ 0 & \lambda_{2} & 0\\ 0 & 0 & \lambda_{3} \end{array}\right]\left[\begin{array}{c} e_{1}^{T}\\ e_{2}^{T}\\ e_{3}^{T} \end{array}\right]$$
  where $\lambda_{1},\lambda_{2},\lambda_{3}$ are the eigenvalues of the neighborhood covariance matrix, and $e_{1},e_{2},e_{3}$ are the corresponding eigenvectors.
3. According to the eigenvalues, the neighborhood feature entropy function $E_{\xi }$ is constructed:
  (3) $$E_{\xi }=-\xi_{1}\ln(\xi_{1})-\xi_{2}\ln(\xi_{2})-\xi_{3}\ln(\xi_{3})$$
  where $\xi_{i}=\frac{\lambda_{i}}{\sum \lambda_{i}}$,i=1,2,3.
4. The adaptive optimal neighborhood radius r_opt_ of point cloud is determined based on the minimum criterion of neighborhood feature entropy function, that is, when $E_{\xi }$ reaches the minimum value, the corresponding neighborhood radius r_j_ is the optimal neighborhood radius r_opt_:
  (4) $$r{\mathrm{opt}}_{\;}=\mathrm{argmin}(E_{\xi })$$

#### 2.3.2. Gaussian Weight-Based Fast Point Feature Histogram

**Figure 3:**
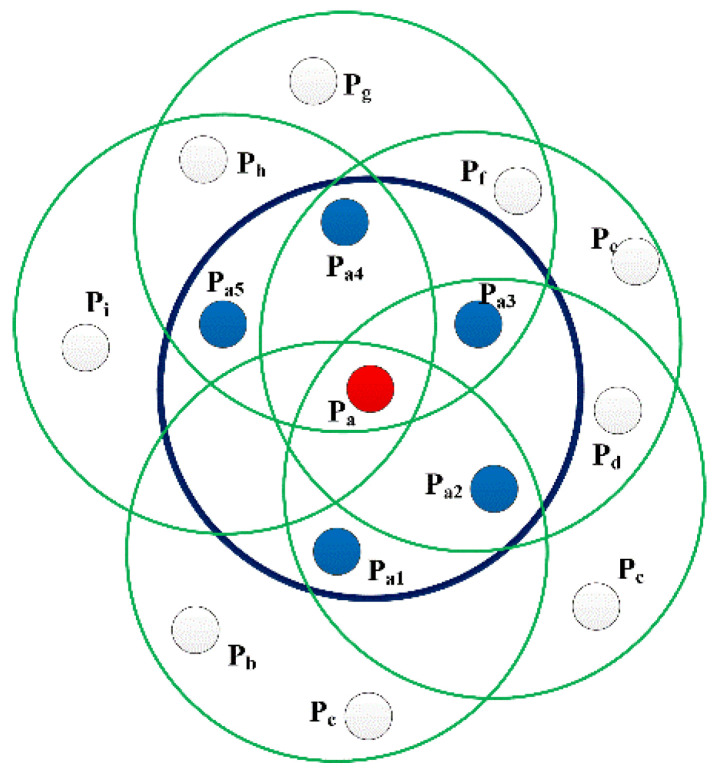
Schematic diagram of FPFH neighborhood.

The features of traditional FPFH are determined by the neighborhood in the radius r of the query point itself and the neighborhood of its neighborhood points, whose maximum range is 2r. The FPFH neighborhood of a query point is shown in Figure 3, where p_a1_–p_a5_ are the neighborhood points of the query point P_a_ within the neighborhood radius r, and p_b_–p_i_ are the neighborhood points of the points p_a1_–p_a5_.

**Figure 4:**
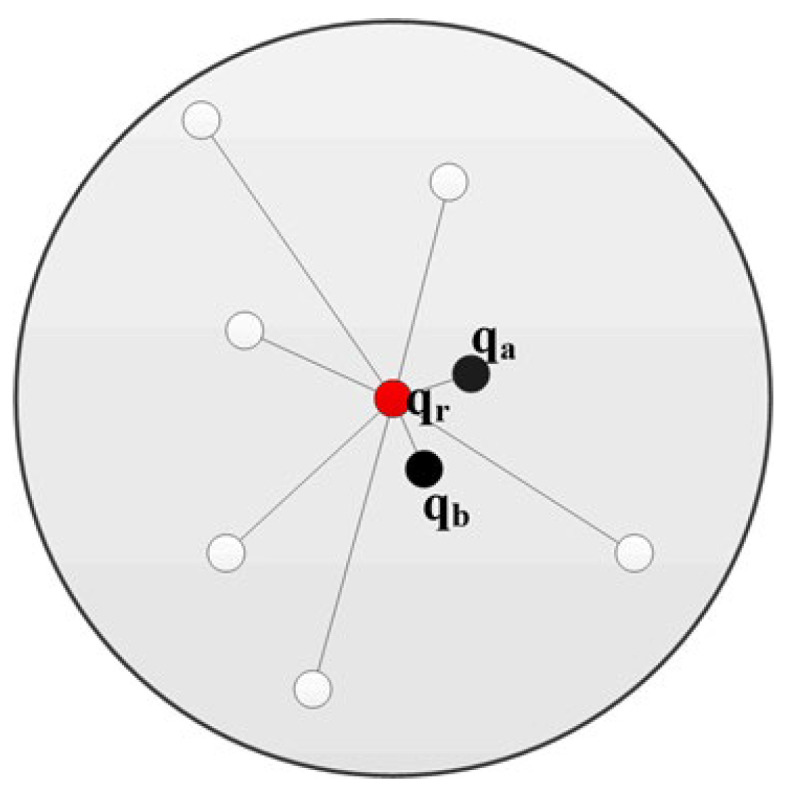
A neighborhood point diagram of a key point.

The weight coefficient of the traditional FPFH algorithm is determined only by the Euclidean distance between the query point and the neighborhoods; often, there are huge differences among various weight coefficients. As is shown in Figure 4, the feature descriptor of the red query point q_r_ is mostly influenced by the closest black points q_a_ and q_b_, and other points only have little effect, which may cause the feature descriptor to be inaccurate.

Therefore, an improved FPFH is proposed; each key point q_k_ in the key point set Q_t_ is taken as the query point, and based on the adaptive optimal neighborhood radius r_opt_, all the neighborhood points q_ki_ within its neighborhood radius are found. The weight coefficient is redefined with the Gaussian distribution, as follows:

(5) $$w_{Gi}=\frac{1}{\sqrt{2\pi }\sigma }\exp(-\frac{{\left(w_{i}-\mu \right)}^{2}}{2\sigma^{2}})$$

where μ is the average distance of q_k_ and q_ki_, and σ represents the standard deviation of the distance between q_k_ and q_ki_.

**Figure 5:**
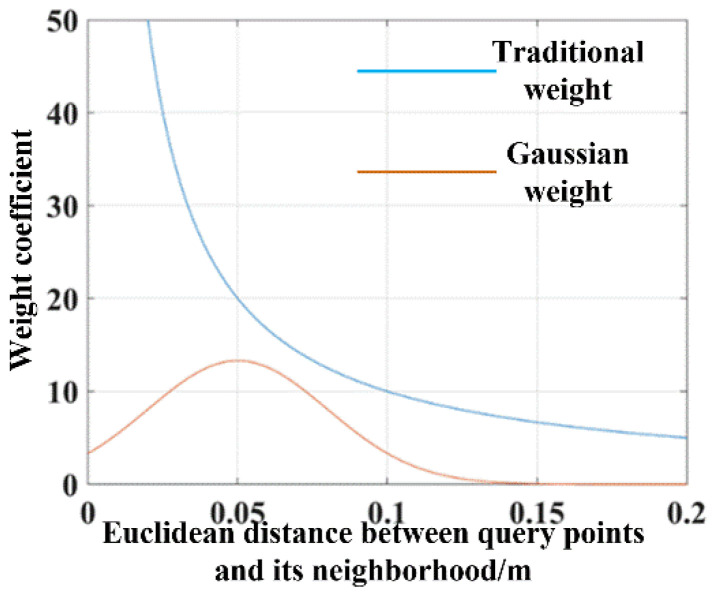
Weight variation trend of neighborhood points of a key point.

The weight coefficient variation trend of the original approach and the proposed approach is shown in Figure 5. It can be seen that the weight coefficient of the original approach varies greatly, and the weight tends to infinity with the decrease of the distance. The proposed AGWF can avoid the problem that the weight coefficient difference between points is apparently large, and the weight coefficient reaches the maximum when the distance is close to the average value, which reasonably solves the unstable problem of the FPFH descriptor calculation.

**Figure 6:**
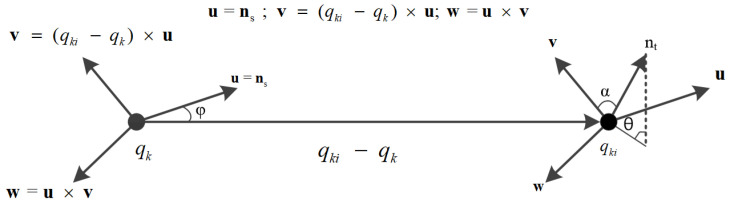
Diagram of local coordinate system (u,v,w).

Combined with the adaptive optimal neighborhood radius selection criterion, an Adaptive Gaussian Weight-based Fast Point Feature Histogram (AGWF) is proposed. It can not only improve the efficiency of feature extraction but also improve the accuracy and robustness; the specific calculation steps are as follows:

1. For each key point q_k_ in the key point set Q_t_, search all the neighborhood points q_ki_ within its optimal neighborhood radius r_opt_.
2. Compute the normal vectors n_s_ and n_t_ corresponding to q_k_ and q_ki_, calculate the relative position deviation (α,ϕ,θ) between n_s_ and n_t_, and generate the Simple Point Feature Histograms (SPFH) of q_k_(SPFH(q_k_)). Local coordinate system (u,v,w) is defined to calculate this deviation, which is shown in Figure 6:

The calculation formula of relative deviation is as follows:

(6) $$\alpha =v\cdot n_{t}\;\varphi =u\cdot \frac{\left(q_{k}-q_{ki}\right)}{{\left\|q_{k}-q_{ki}\right\|}_{2}}\;\theta =\arctan(w\cdot n_{t},u\cdot n_{t})$$

3. Then, search the neighborhood points of q_ki_ based on the adaptive optimal neighborhood radius r_opt_, and generate the SPFH of q_ki_(SPFH(q_ki_)). Based on the Gaussian weight w_Gi_, the SPFH(q_ki_) is weighted to obtain the Adaptive Gaussian Weight-based Fast Point Feature Histogram of key points q_k_(AGWF(q_k_)):

(7) $$AGWFPFH(q_{k})=SPFH(q_{k})+\frac{1}{k}\sum_{i=1}^{k}w_{Gi}SPFH(q_{ki})$$

### 2.4. Point Cloud Registration

#### 2.4.1. Coarse Registration

The purpose of coarse registration is to obtain the initial pose relationship between the source point cloud P_t_ and the target point cloud, so as to overcome the shortcomings of the ICP algorithm, which requires high initial pose and is easy to fall into local optimum. The SAC-IA algorithm can effectively adjust the initial pose relationship between the source point cloud P and the target point cloud Q and improve the accuracy of pose estimation. The specific steps are as follows:

1. Compute AGWF feature descriptors for all key points in the source point cloud P and the target point cloud Q.
2. N sample points P_u_ (u = 1, 2, …, N) are randomly selected from the source point cloud P, and the distance between two sample points is greater than the preset distance threshold d_min_.
3. According to the AGWF, search the closest points Q_u_ (u = 1, 2, …, N) in the target point cloud Q to the sample points P_u_, and obtain the initial match point pairs.
4. Obtain the rigid transformation matrix **M**_1_ between initial match point pairs by Singular Value Decomposition (SVD). Set a registration threshold e_l_, and calculate the distance function H(l_i_) to evaluate the point cloud registration performance. The expression of the distance function H(l_i_) is as follows:
  (8) $$H(l_{i})=\left\{ \begin{array}{ll} \frac{1}{2}l_{i}^{2} & {\left\|l\right\|}_{i}<e_{l}\\ \frac{1}{2}e_{l}(2{\left\|l\right\|}_{i}-e_{l}) & {\left\|l\right\|}_{i}\geq e_{l} \end{array}\right.$$
  where l is the average Euclidean distance between the source point cloud, and I is the number of iterations.
5. Repeat the above four steps; when H(l_i_) reaches the minimum, the corresponding transformation matrix is the coarse registration rigid transformation matrix **M**_c_. The rigid transformation of the source point cloud P is carried out based on **M**_c_ to obtain the point cloud P_r_, and coarse registration is completed.

#### 2.4.2. Accurate Registration

After coarse registration, the source point cloud Pr and the target point cloud Q can only roughly coincide, so it is necessary to improve the pose estimation accuracy by further accurate registration. The ICP algorithm is used for accurate registration. The algorithm obtains the nearest Euclidean point through exhaustive search and obtains accurate registration parameters based on the results of the optimal objective function. According to the registration parameters, 6 Degrees of Freedom (6 DOF) pose estimation result can be obtained [33].

1. Set a distance threshold e_f_ and the maximum number of iterations I_0_. For each point p_ri_ in the source point set P_r_, search for its corresponding closest point q_i_ in the target point set Q, and form the corresponding points pairs, set C_l_.
2. Solve the rigid transformation matrix by SVD and obtain the rotation matrix **R**_n_ and the translation matrix **T**_n_, where n is the number of iterations. Convert the source point P_r_ by the translation matrix (**R**_n_, **T**_n_) into P_rn_, and form the corresponding point pairs C_n_. Calculate the average Euclidean distance e_n_ between every corresponding point pair.
  (9) $$e_{n}=\frac{1}{k}{\sum_{i=1}^{k}\left\|q_{i}-(p_{ri}\times R_{n}+T_{n})\right\|}^{2}$$
  where k is the number of the corresponding point pairs.
3. Repeat the above steps until e_n_ is smaller than e_f_ or the maximum number of iterations I_0_ is reached, and finally obtain the optimal transformation matrix **R** and **T**.
4. Let **R**_x_, **R**_y_ and **R**_z_ be the rotation angles of the three coordinate axes, and **t**_x_, **t**_y_ and **t**_z_ be the translation vectors of the coordinate axes; the 6 DOF pose estimation can be represented as (**R**_x_, **R**_y_, **R**_z_, **t**_x_, **t**_y_, **t**_z_). The optimal transformation matrix **M**_a_ can be expressed as

(10) $$M_{a}=T(t_{x},t_{y},t_{z})\times R(R_{x},R_{y},R_{z})$$

where $T(t_{x},t_{y},t_{z})$ and $R(R_{x},R_{y},R_{z})$ can be expressed as

(11) $$T(tx,ty,tz)=\left(\begin{array}{cccc} 1 & 0 & 0 & tx\\ 0 & 1 & 0 & ty\\ 0 & 0 & 1 & tz\\ 0 & 0 & 0 & 1 \end{array}\right)$$

(12) $$R(R_{x},R_{y},R_{z})=\left(\begin{array}{cccc} r_{11} & r_{12} & r_{13} & 0\\ r_{21} & r_{22} & r_{23} & 0\\ r_{31} & r_{32} & r_{33} & 0\\ 0 & 0 & 0 & 1 \end{array}\right)$$

It can be concluded that the 6DOF pose estimation parameters of the target can be expressed as

(13) $$\left(R_{x},R_{y},R_{z},t_{x},t_{y},t_{z})=(\arctan(r_{32}/r_{33}),\arcsin(-r_{13}),\arctan(r_{21}/r_{11}),t_{x},t_{y},t_{z}\right)$$

According to the actual situation of pallet fork taking in the storage environment, only the horizontal deviation t_x_ and t_z_ and the rotation angle R_y_ perpendicular to the ground need to be obtained. The driverless industrial trucks adjust the pallet fork taking path according to the deviation parameters (t_x_, t_y_, R_y_).

## 3. Pallet Pose Estimation Experiment

### 3.1. Data Collection

**Figure 7:**
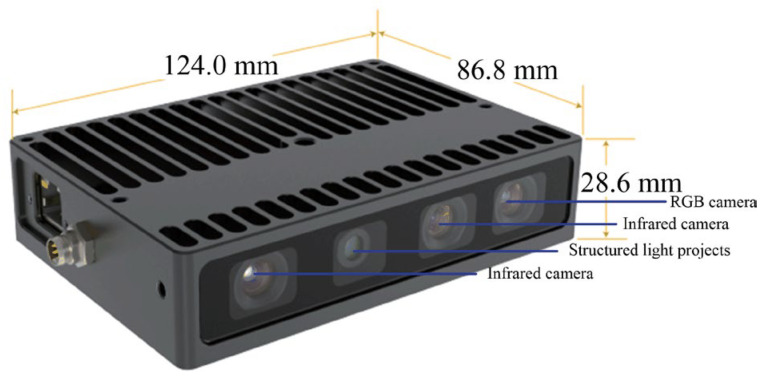
Structure of the Percipio FM851-E2 vision sensor.

An industrial vision sensor called the Percipio FM851-E2 3D vision sensor is adopted to acquire point source point cloud and scene point cloud, whose ranging principal is active binocular, and the operative range is 0.7–6.0 m. The structure of the Percipio FM851-E2 vision sensor is shown in Figure 7.

**Figure 8:**
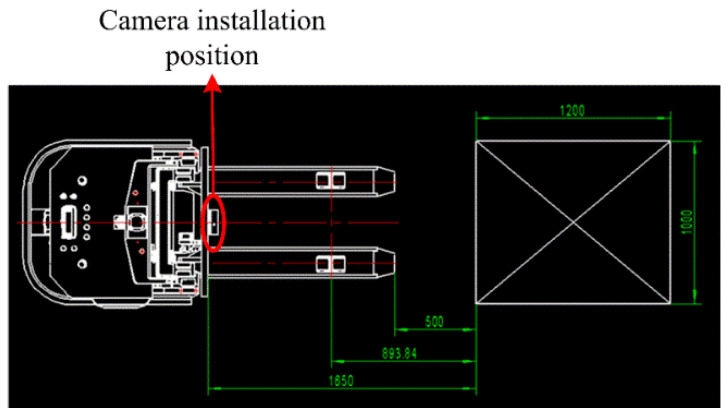
Relative position of unmanned industrial vehicle and pallet in standard state.

**Figure 9:**
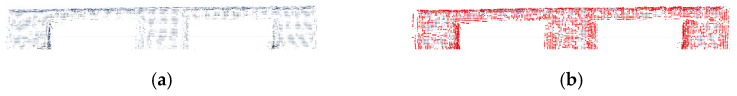
(**a**) The source point cloud. (**b**) The key points of source point cloud.

**Figure 10:**
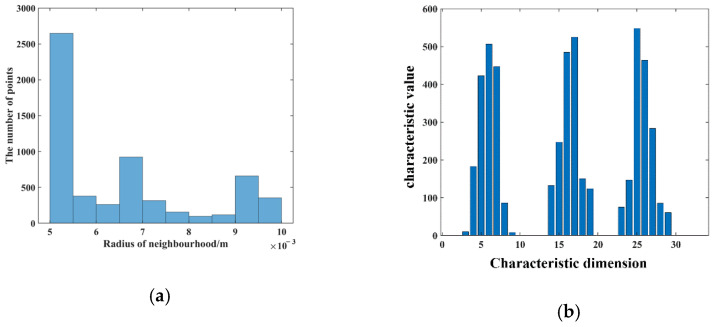
Processing results of source point cloud. (**a**) Adaptive optimal neighborhood distribution. (**b**) The AGWF feature descriptor.

To obtain the source point cloud of the pallet, a normal blue pallet is placed in a fixed position in a laboratory with normal brightness and no other obstructions, and the Percipio FM851-E2 vision sensor is fixed on the top of the fork frame to take pictures as shown in Figure 8. To meet the operational requirements of the production floor, the front of the fork is placed 500 mm away from the pallet, ensuring that the fork is perpendicular to the front of the pallet and the center of the sensor is aligned with the center of the pallet. The collected point cloud is processed by the point cloud filter and plain segmentation algorithm, and the remaining points are the source point cloud, which contains the position information of the pallet point cloud in the sensor coordinate system under the standard state after visualization, as shown in Figure 9. The key points of the source point cloud are extracted, and the adaptive optimal neighborhood radius is obtained; then, the AGWF features are calculated (the results are shown in Figure 10), and the results are saved in the database.

### 3.2. Multiple Scenario Experiments

In order to verify the effectiveness of the pose estimation algorithm, the pallet pose estimation algorithm is experimentally verified in several scenarios, mainly the common ground scenarios and shelf scenarios.

#### 3.2.1. The Ground Scene

**Figure 11:**
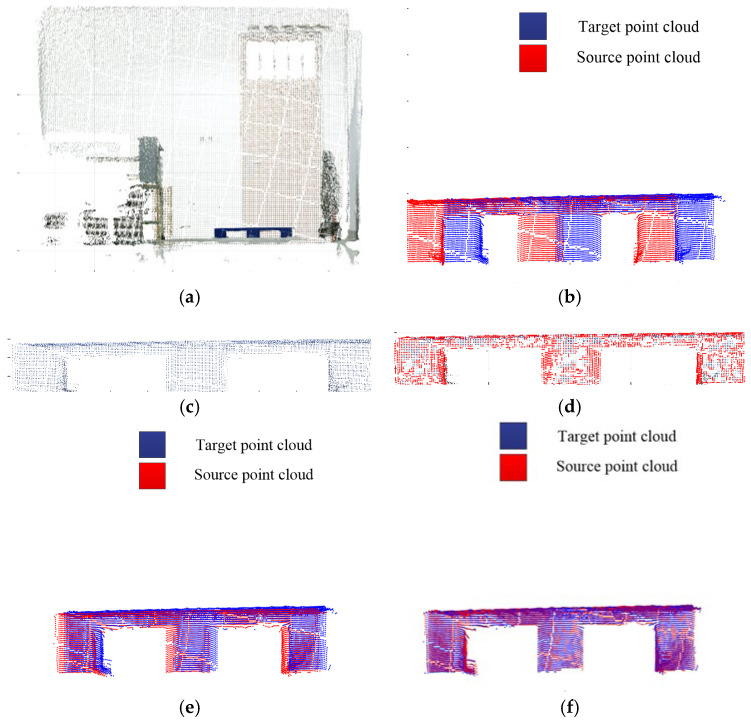
Point cloud processing results of 5° deflection angle. (**a**) Scene point cloud. (**b**) Initial relative position. (**c**) Target point cloud. (**d**) Key points of the target point cloud. (**e**) Coarse registration. (**f**) Accurate registration.

Considering the practical requirements of the pallet attitude estimation scenario, the relative attitude relationship between the storage pallet and the sensor is considered only for the horizontal lateral translation Δx, the longitudinal translation Δy, and the deflection angle φ. The deflection angles of 5°, 10°, 15° and 20° and the deviations of 0.05 m, 0.10 m, 0.15 m and 0.2 m in the horizontal direction, respectively, are selected for the experiments. The correction of the deflection angle needs to be completed through the rotation of the driverless industrial truck, so the rotation center of the deflection angle is actually the origin of the camera coordinates in this experiment. The scene point clouds are taken and preprocessed, the target pallet point clouds in the scene point clouds are extracted, and the adaptive optimal neighborhoods of key points and AGWF feature descriptors are calculated to match with the pallet point clouds. Taking the experiment at a deflection angle of 5° as an example, the visualization results of the scene point cloud, the relative position relationship between the target point cloud and the source point cloud, the key points of the target point cloud, and the rough and accurate registration are listed in Figure 11.

**Figure 12:**
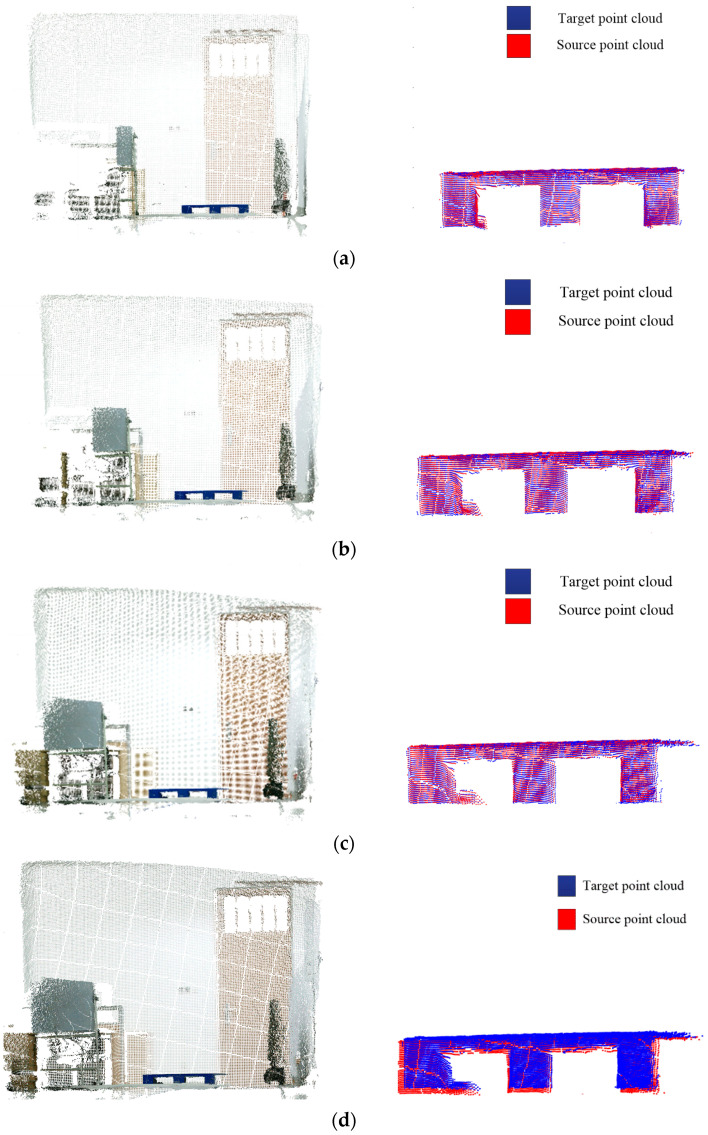

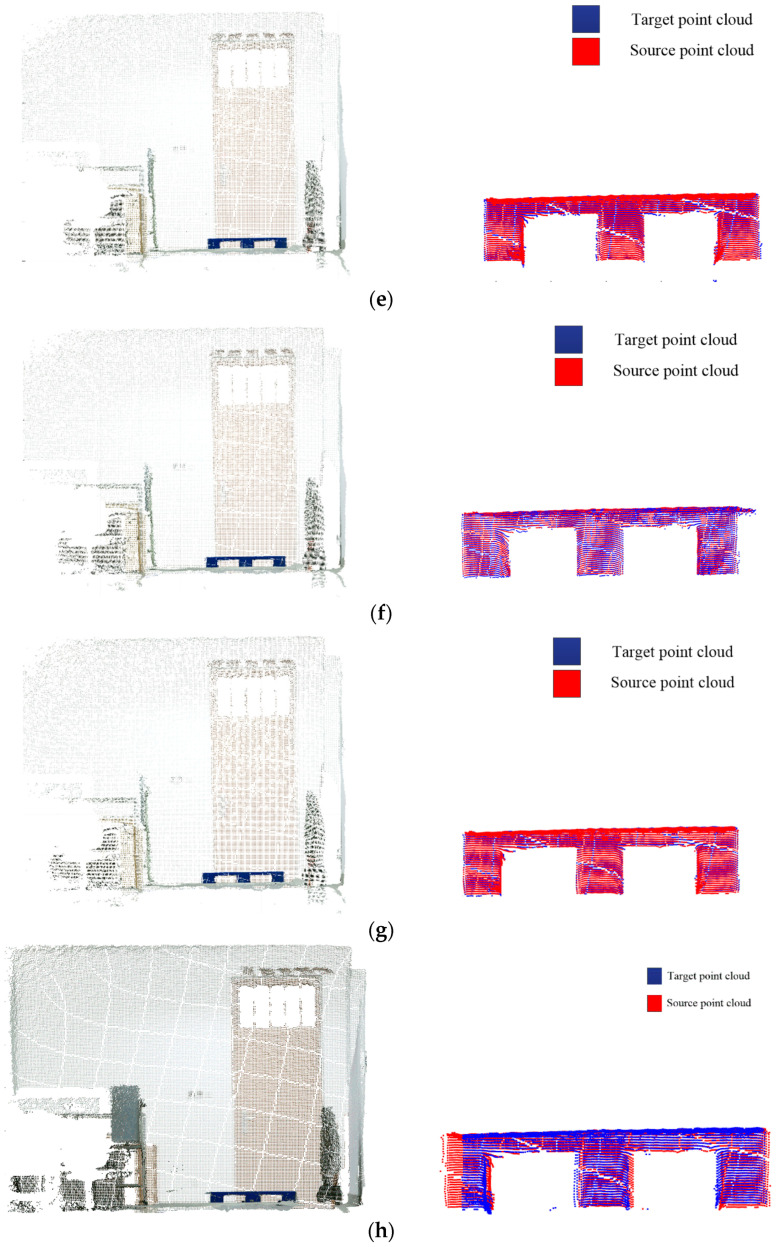
Scene point cloud and registration result. (**a**) 5° deflection angle. (**b**) 10° deflection angle. (**c**) 15° deflection angle. (**d**) 20° deflection angle. (**e**) Deviations of 0.05 m. (**f**) Deviations of 0.10 m. (**g**) Deviations of 0.15 m. (**h**) Deviations of 0.2 m.

The number of point clouds and the time consumed for each step of the experiments are shown in Table 1, and the scene point clouds captured and the registration results are shown in Figure 12. The experiments’ pose estimation results and errors are shown in Table 2. The experiments (d) and (h) are the experimental control groups with the out-of-limit deviations (bolded in Table 2), which are not taken into account for calculating the total average deviation and accuracy.

As can be seen from the experimental results derived from the above table, the average estimation error of the horizontal direction is about 0.0098 m, the average estimation error is about 0.0194 m, and the average estimation error of the deflection angle is about 0.5°, with a total average accuracy of 97.3%. It can be seen that the algorithm has high accuracy and strong robustness when the horizontal deviation is within 0.15 m and the deflection angle is within 15°. However, as is shown the experiments (d) and (h) in Figure 12, when the horizontal deviation or deflection angle is too large, due to the field of view limitation of the depth camera and excessive initial pose deviation, the target point cloud and the source point cloud may fail to be aligned, which will affect the accuracy of the pallet pose estimation.

#### 3.2.2. The Shelf Scene

In a production workshop that has several shelves with three layers, a blue pallet is placed in the second layer, which has a certain position deviation with the standard state. The driverless industrial truck follows a preset path to a designated location, and the horizontal deflection and the size of the deflection angle must be calculated by the pose estimation algorithm to realize the accurate forklift of pallets for driverless industrial trucks.

**Figure 13:**
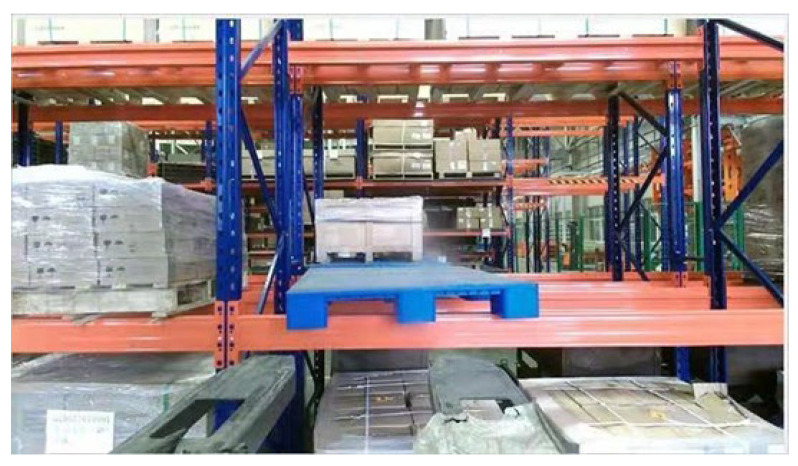
Color image of the shelf.

**Figure 14:**
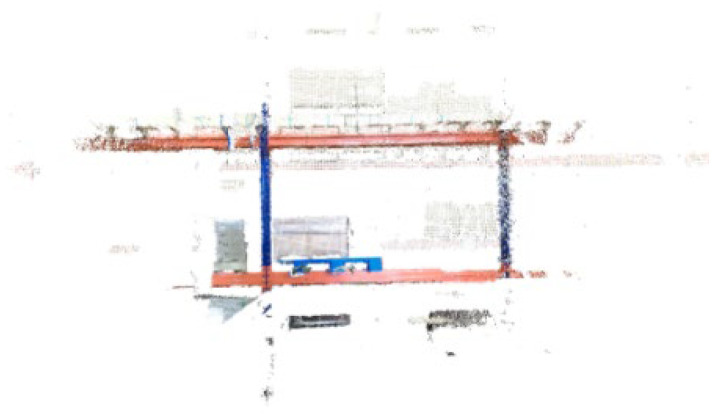
Shelf scene point cloud.

**Figure 15:**
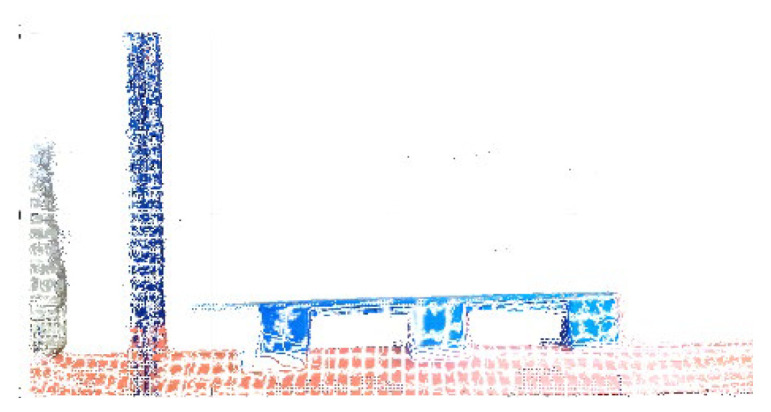
Pass-through filtering.

**Figure 16:**
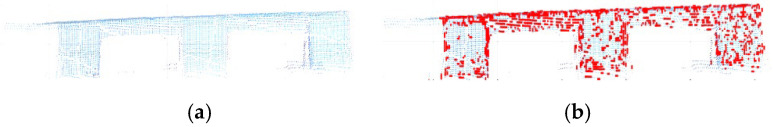
Key point extraction for target point cloud. (**a**) Target pallet point cloud. (**b**) Target pallet key points.

The color image of the scene captured by the Percipio active binocular vision sensor is shown in Figure 13, and the scene point cloud is shown in Figure 14, among which the number of the scene points is 2,073,600. Redundant points were eliminated by pass-through filtering. According to the position relationship between the vision sensor and the pallet, the pass-through filtering parameters x, y and z are set as x∈(−1.0,1.0) m, y∈(−0.5,0.5) m, z∈(1.5,3.0) m to control the filtering interval of point cloud, and the number of remaining point clouds is 259,443; the results are shown in Figure 15. After plane segmentation and voxel grid downsampling, the target pallet point cloud in this shelf scene is obtained, and the remaining point cloud number is 7438, which is shown in Figure 16a. The search radius of key point extraction is set as 0.05 m, and the two thresholds are set as r_1_ = 0.4 and r_2_ = 0.2. A total of 1476 key points are extracted from the target point cloud, as shown in Figure 16b, the red points are the key points.

**Figure 17:**
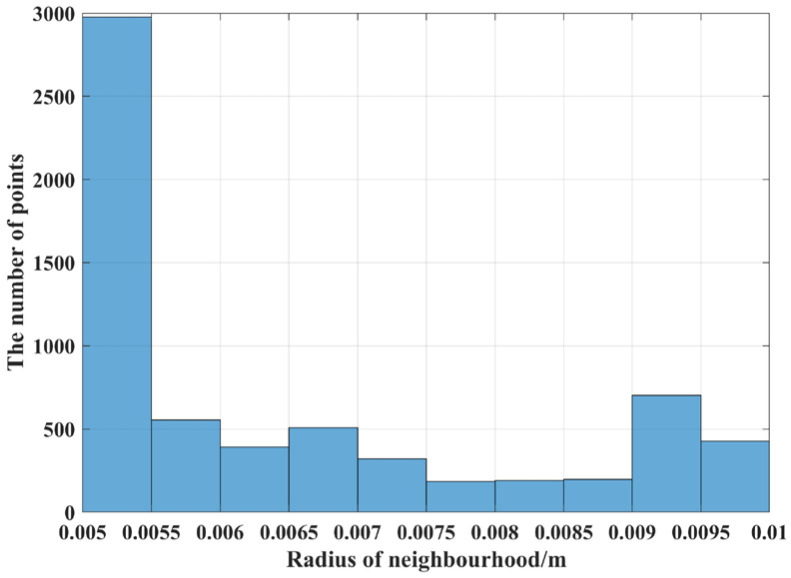
Shelf scene target pallet point cloud adaptive optimal neighborhood radius.

Before computing the AGWF feature descriptors for the target point cloud, the adaptive neighborhood radius of each point needs to be determined and set to r_min_ = 0.006 m, r_max_ = 0.012 m, and r_d_ = 0.001 m, and the average distance d_p_ = 0.006 m of the target point cloud is calculated. The adaptive optimal neighborhood radius of each point is obtained according to the minimum criterion of the neighborhood characteristic entropy function, and the adaptive optimal neighborhood radius distribution is shown in Figure 17. The horizontal coordinate indicates the different neighborhood radii, and the vertical coordinate indicates the number of points corresponding to each neighborhood radius. It can be seen that the optimal neighborhood radius of key points is concentrated on the given minimum neighborhood radius, which can significantly improve the efficiency of the feature descriptor.

**Figure 18:**
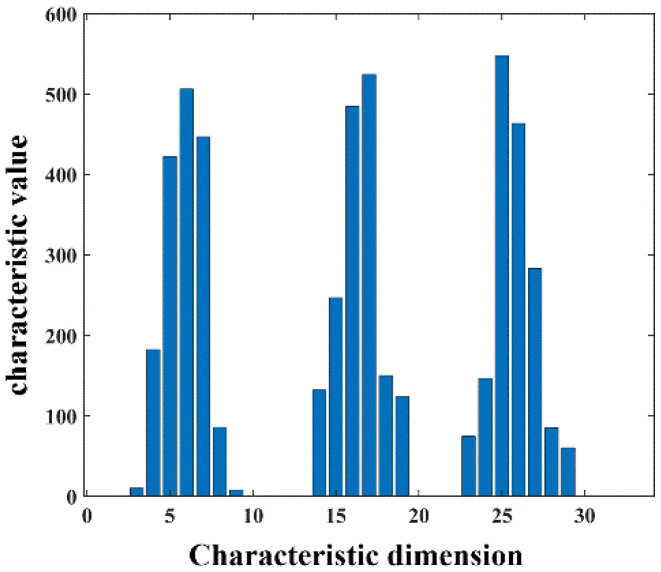
The AGWF feature descriptor for a point.

The AGWF feature descriptors of the source point cloud and the target pallet point cloud are calculated based on the minimum neighborhood radius, and the AGWF feature of a point is shown in Figure 18. 

The SAC-IA algorithm is used for the coarse registration, and the ICP is used to complete the accurate registration; the accurate optimal rigid rotation matrix **R** and the translation matrix T are obtained as follows:

$$R=\left[\begin{array}{ccc} 0.99221826 & -0.0056165964 & -0.12438513\\ 0.0041089170 & 0.99991482 & -0.012374031\\ 0.12444409 & 0.011766853 & 0.99215692 \end{array}\right]$$

$$T=\left[\begin{array}{l} 0.35200304\\ -0.083008856\\ 0.018321276 \end{array}\right]$$

**Figure 19:**
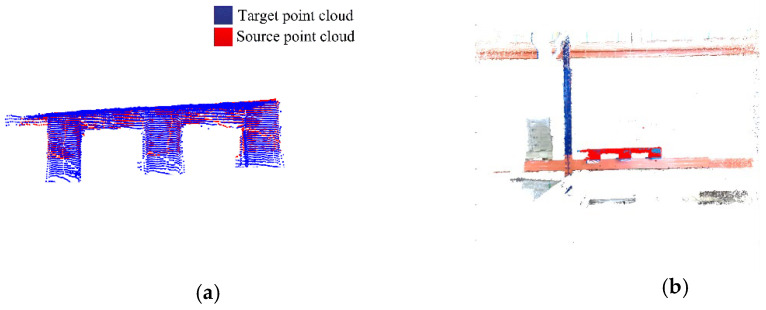
Visualization of pallet pose estimation results. (**a**) Registration result (**b**) Pallet pose estimation.

The result of the accurate registration is shown in Figure 19. The left figure shows the registration diagram of the source point cloud and the target pallet point cloud, where the red point cloud is the source point cloud and the blue point cloud is the pallet source point cloud. The figure on the right shows the pose of the source point cloud after transformation according to the accurate optimal rigid transformation matrix, where the red point cloud is the source point cloud, and the rest of the point clouds are the scene point clouds captured by the Percipio vision sensor while the driverless industrial truck is working. As can be seen in Figure 19, after the rigid transformation, the source point cloud and the target pallet point cloud in the field point cloud can achieve basic overlap, so it can be considered that the rotation matrix R and translation matrix T obtained from the accurate registration is reliable, and the source point cloud and the target point cloud have satisfactory registration results, which further verifies the accuracy of the proposed pose estimation algorithm. According to the rotation matrix R and the translation matrix T, it can be calculated that the horizontal deviations Δx and Δy are 0.35 m and 0.018, respectively, the deflection angel is 12.43°, and the pose estimation is completed.

### 3.3. Results and Discussion

**Figure 20:**
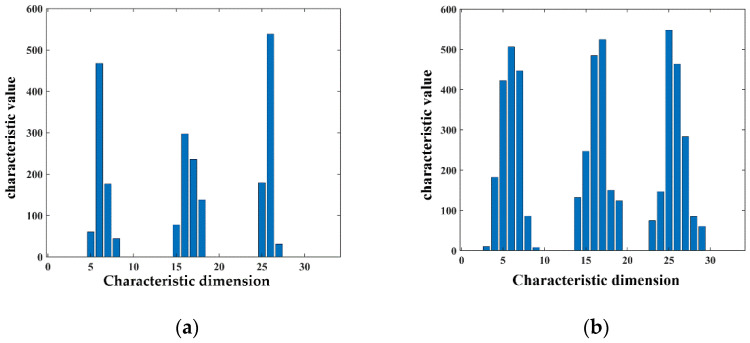
Comparison of FPFH and AGWF at a point. (**a**) The FPFH feature descriptor for a point. (**b**) The AGWF feature descriptor for a point.

To verify the rationality of Adaptive Gaussian Weight-based Fast Point Feature Histogram, a comparison is made with the traditional FPFH feature descriptor. A diagram of the comparison between AGWF and FPFH for a point is shown in Figure 20, with the horizontal coordinates indicating the feature dimensions and the vertical coordinates indicating the feature values on each dimension.

**Figure 21:**
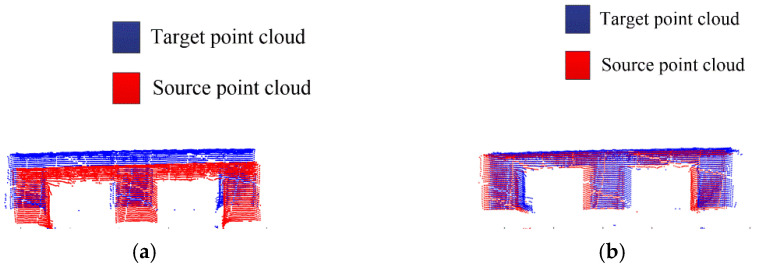
Comparison of coarse registration results between FPFH and AGWF. (**a**) Coarse registration based on FPFH. (**b**) Coarse registration based on AGWF.

As can be seen in Figure 20, the proposed AGWF descriptor has more balanced values among dimensions and can describe more feature information as compared with the FPFH feature descriptor. The problem that the feature descriptor is overly influenced by a certain neighborhood point is avoided, and the robustness of the feature descriptor is improved. The comparison of coarse registration results between FPFH and AGWF is shown in Figure 21.

The proposed approach is compared with the traditional PFH and FPFH feature descriptors as well as the advanced SHOT feature descriptors in terms of the feature extraction time consumption and the RMSE. The experimental results of different descriptors are shown in Table 3, where R is the neighborhood radius used for feature extraction, T is the time consumed for feature extraction, Dt is the percentage of time consumption reduction of the proposed method compared with the traditional methods, DRMSE is the percentage of error reduction of the proposed method compared with the traditional methods, and GWF is Gaussian Weight-based Fast Point Feature Histogram, which only improves the traditional FPFH by Gaussian weight without adaptive neighborhood. In Table 4, the neighborhood radius of the traditional descriptors is set as 0.014 m, and the neighborhood radius of the AGWF is set adaptively. The GWF and AGWF are the proposed feature descriptors in this paper, and they are bolded in the Table 3 and 4.

As is shown in Table 3, the time consumption of the GWF is 7.9% and 17.34% longer than the SHOT and FPFH, respectively, and it is 56.6% faster than the PFH. Compared with the PFH, FPFH and SHOT, the RMSE of the GWF is reduced by 10.99%, 54.47% and 7.57%, respectively. The GWF does not have an absolute advantage in terms of time, because it needs to consider more information during feature extraction, but it has higher registration accuracy. It can be seen in Table 4, when the neighborhood radius is set to 0.014 m, the time consumption of the proposed method is reduced by 46.61%, 79.16% and 48.15%, and the RMSE is reduced by 36.35%, 68.75% and 36.23%, respectively. Comparing Table 3 and Table 4, the choice of the neighborhood radius can influence the time consumption of the feature extraction and the accuracy of the point cloud registration, and the proposed adaptive radius can significantly reduce the time consumption of feature extraction and the accuracy of the point cloud registration. Compared with the fixed radius (GWF), the proposed AGWF feature descriptor reduces the time consumption by 43.27%, and the RMSE by 27.86%. Above all, the proposed method can reduce the time consumption by more than 30% and the error by more than 35%.

Compared with the traditional FPFH feature descriptor, the proposed AGWF feature descriptor improves the weight coefficients in its calculation process, which can describe the local features of the point cloud better and fully consider the influence of each neighboring point on the key point features, thereby improving the accuracy and efficiency of the bit pose estimation. In addition, the methods based on traditional descriptors usually set the search radius of feature descriptors through experience when setting the radius of the key point neighborhood, which has the problem of inefficiency. The proposed approach adaptively sets the neighborhood radius according to the minimum criterion of the neighborhood information entropy function, which greatly reduces the computation time of feature descriptors and achieves efficient and high-precision pose estimation of the pallet.

## 4. Conclusions

A point cloud data-driven pallet pose estimation method using an active binocular vision sensor is proposed, which consists of point cloud preprocessing, adaptive Gaussian weight-based fast point feature histogram definition and point cloud registration, and improves the pose estimation accuracy by over 35% and reduces the feature extraction time by over 30%. The main contributions of the proposed method can be summarized as follows:

1. A point cloud-driven method for the driverless industrial trucks to estimate the pose of the pallet in the production shop is proposed, which solves the problem that the pallet cannot be accurately forked due to the position deviation, and improves the security and stability of the logistics system.
2. An adaptive optimal neighborhood radius selection criterion based on the minimum rule of the local neighborhood characteristic entropy function is proposed to determine the neighborhood radius of each key point adaptively, instead of selecting parameters based on experience manually, which significantly shortened the time of feature extraction and improved the accuracy.
3. Traditional descriptors only consider the Euclidean distance between the query key point and the neighborhood point as the traditional methods, and the weight of the proposed descriptor is optimized by the Gaussian distribution function. The change of the weights of each neighborhood point is smoother and can describe the key point features more accurately and completely, thereby effectively improving the robustness of the feature descriptors.

Nevertheless, since the proposed approach still requires a large amount of computation on the point cloud data, the real-time performance of the approach needs to be further improved. At the same time, due to the complex storage environment, the proposed approach also needs further discussion on problems such as vision occlusion and multi-target overlap caused by dynamic and static obstacles. The subsequent work will focus on improving the real-time performance of the algorithm, as well as research on scenes with different lighting and different degrees of occlusion. 

## Figures and Tables

**Table 1:** Point cloud processing process.

Serial No.	Number of Scene Point Clouds	Number of Target Point Clouds	Number Of Key Points	Time Consumed/s
a	55,827	6181	1154	1.6080
b	58,788	6500	1327	1.8263
c	64,656	7115	1442	1.9777
d	53,433	7155	1548	1.3595
e	50,085	5533	1014	0.7670
f	47,142	5217	1272	1.2597
g	44,118	4895	916	1.2393
h	36,054	4001	1173	1.0132

**Table 2:** Experimental results of the ground scene.

Serial No.	Actual Deviation			Estimated Deviation			Relative Deviation			Accuracy/%
	Δx	Δy	φ	Δx	Δy	φ	Δx	Δy	φ	
a	0	0	5	0.0102	0.0267	4.8876	0.0102	0.0267	0.1124	97.76
b	0	0	10	0.0109	0.0340	9.8656	0.0109	0.0340	0.1344	98.66
c	0	0	15	0.0212	0.0534	15.09	0.0212	0.0534	0.1900	98.73
**d**	**0**	**0**	**20**	**0.0536**	**0.0687**	**26.53**	**0.0536**	**0.0687**	**6.5300**	**67.30**
e	0.050	0.050	0	0.0535	0.0505	0.4060	0.0035	0.0005	0.4060	96.00
f	0.100	0.100	0	0.1096	0.0988	1.0620	0.0096	0.0012	1.0620	94.60
g	0.150	0.150	0	0.1536	0.1477	1.2014	0.0036	0.0023	1.2014	98.03
**h**	**0.200**	**0.200**	**0**	**0.1928**	**0.1579**	**3.2503**	**0.0072**	**0.0421**	**3.2503**	**87.68**
Total Average							0.0098	0.0194	0.5010	97.30

**Table 3:** Comparison of experimental results of different descriptors (R = 0.012 m).

Names	R/m	T/s	RMSE	Dt/%	DRMSE/%
SHOT	0.012	1.564	0.018234	−7.9	10.99
PFH	0.012	3.872	0.035647	56.6	54.47
FPFH	0.012	1.430	0.017559	−17.34	7.57
**GWF**	**0.012**	**1.678**	**0.016239**	**/**	**/**

The Dt and DRMSE are calculated as Dt = (FT − GWF)/FT, DRMSE = (FR − GWF)/FR, where FT is the feature extraction time of the traditional descriptors, and FR is the RMSE of traditional descriptors.

**Table 4:** Comparison of experimental results of different descriptors (R = 0.014 m).

Names	R/m	T/s	RMSE	Dt/%	DRMSE/%
SHOT	0.014	1.564	0.018234	46.61	36.35
PFH	0.014	3.872	0.035647	79.16	68.75
FPFH	0.014	1.430	0.017559	48.15	36.23
**AGWF**	**Adaptive radius**	**0.952**	**0.011715**	**/**	**/**

The Dt and DRMSE are calculated as Dt = (FT − AGWF)/FT, DRMSE = (FR − AGWF)/FR, where FT is the feature extraction time of the traditional descriptors, and FR is the RMSE of traditional descriptors.

## Data Availability

Not applicable.
